# Revision of the genus *Heteranassa* Smith, 1899 (Lepidoptera, Erebidae, Omopterini)

**DOI:** 10.3897/zookeys.527.8771

**Published:** 2015-10-15

**Authors:** Nicholas Homziak, Heidi Hopkins, Kelly B. Miller

**Affiliations:** 1 Kelly Miller Lab, Department of Biology, University of New Mexico, 167 Castetter Hall, MSC03 2020, Albuquerque, NM 87131–0001 USA; 2 Current address: Department of Entomology, University of Florida, P.O. Box 110620, Gainesville, FL 32611–0620 USA

**Keywords:** *Prosopis*, Omopterini, *Heteranassa*, Mesquite, *Acacia*

## Abstract

*Heteranassa* Smith (Erebidae, Omopterini), native to the southwestern United States and Mexico, includes two recognized species, namely *Heteranassa mima* (Harvey) and *Heteranassa fraterna* Smith. These are separated mainly by subtle differences in wing color and pattern, leading to speculation about the validity of the described species. This study examines variation in external and internal morphology across the geographic range of the genus, aiming to clarify species limits, describe morphology, and provide a comprehensive assessment of variation within the genus. Results indicate that *Heteranassa fraterna* **syn. n.**, is a junior synonym of *Heteranassa mima*.

## Introduction

*Heteranassa* Smith, 1899, is a genus of moths native to warmer desert regions of southwestern United States southward to southern Mexico, currently containing two valid species. *Heteranassa mima* (Harvey, 1876), described from Texas, and *Heteranassa fraterna* Smith, 1899, described from Death Valley, California. Mustelin (2006) synonymized an additional species, *Heteranassa minor* Smith, 1899, with *Heteranassa fraterna*. *Heteranassa* feed on mesquite (*Prosopis* sp.) and Acacia (*Acacia* sp.) (both Fabaceae) and are multivoltine (Crumb 1956), with adults occurring year round.

*Heteranassa* species show a range of wing pattern variation within series collected at the same locality, but also seem to exhibit some geographic variation. To aid in the identification of these common moths, this study assesses the number of species in *Heteranassa*, clarifies the nomenclature, provide detailed descriptions of the adults and larvae, and document the phenotypic variation.

## Methods

Specimens were collected in Death Valley, Inyo Co., California, (February 2005), White Sands National Monument, Otero Co., New Mexico, (August 2010, 2011), Cuatrocienagas Protected Area, Cuatrociénagas, Coahuila, Mexico, (June, September 2011), Pima Co., Arizona, (July 2012), and Socorro Co., New Mexico (October 2012). Specimens were collected with a sheet trap using 15W UV fluorescent lamp, 175W Mercury Vapor lamp, or a 175W, 6500K metal halide lamp. Death Valley specimens were collected at incandescent or fluorescent outdoor lighting at the Furnace Creek Ranch Hotel.

Specimen loans were generously provided by the following institutions:

UASM University of Alberta Strickland Entomology Museum, Edmonton, Alberta (F.A.H. Sperling)

EMEC Essig Entomology Museum, University of California, Berkeley (J. Powell)

LACM Natural History Museum of Los Angeles County, Los Angeles, California (B. Brown)

UAIC University of Arizona Insect Collection, Tucson (W. Moore)

ASUT Arizona State University Entomology Collection, Tempe (T. Dowling)

CUIC Cornell University Insect Collection, Ithaca, New York (J. Liebherr)

KSUC Kansas State Entomological Museum, Manhattan (G. Zolnerowich)

Specimens were also examined during visits to the United States National Museum (USNM, M. Pogue) and the McGuire Center for Lepidoptera and Biodiversity (MGCL, A. Warren). A complete list of specimens examined is included in Suppl. material 1. Dissected material was selected to represent the range of size and coloration found across the range of *Heteranassa*. A list of dissected specimens is included in Suppl. material 2.

**Figure 1.**
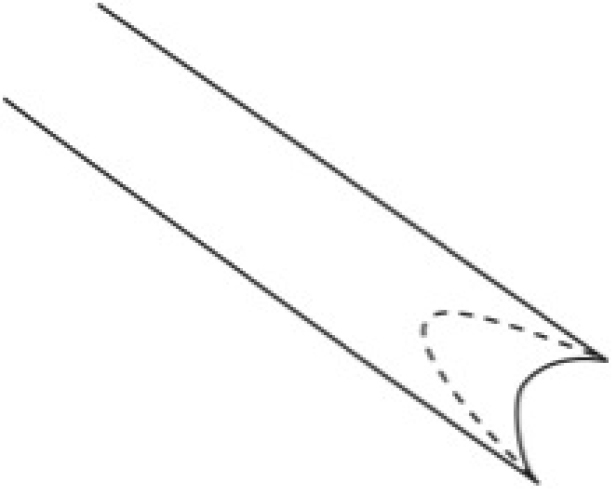
30 Gauge syringe modified to fit over aedeagus.

Genitalic dissections follow techniques described by Hardwick (1950) and McCabe (1980). Terminology follows Forbes (1923, 1954) and Mikkola et al. (2009). Abdomens were removed from specimens by gently applying upward pressure near the end of the abdomen with a pair of angle–tipped forceps. Abdomens were cleared in 10% KOH overnight or in hot KOH for 15 minutes. The abdomens were then placed in a watch glass with distilled water, and scales were removed with a fine brush. Once clear of scales, the integument of the abdomen was cut along the left pleural membrane, and the genital capsule removed. On male specimens setae were carefully removed from the membranous costal region of valves with a fine camel’s hair brush. The aedeagus was separated from the valves by grasping the distal end with fine–tipped forceps, and gently pulling to separate from the juxta. The ductus seminalis was then cut where it enters the side of the proximal part of the aedeagus. The vesica was then carefully teased out of the aedeagus with a #20 minuten with the tip bent to a right angle, held in a standard pin vice, and with water pressure from a syringe. A syringe with a modified 30 gauge needle (Fig. 1) was used to force water into the opening of the ductus seminalis to help evert and inflate the vesica. The aedeagus was transferred to 95% ETOH to dehydrate. The vesica was inflated with ETOH, following the procedures described by McCabe (1980), for several seconds using the modified syringe. The valves were transferred to 95% ETOH, and the membranous lobes of the sacculus were inflated with 95% ETOH. Abdomens of female specimens were cleared in hot KOH. The abdomen was cut along the left pleural membrane, and then a circular incision was made around abdominal segment VIII to remove the female genitalia. Genitalia dissections were placed in Chlorazol Black® stain for ~10 seconds, then transferred to 95% ETOH to dehydrate overnight. The genitalia were placed overnight in orcein stain dissolved in 2–propanol. Genitalia and abdomen “pelts” were stored in pin–mounted glycerin vials. Material from the first author’s personal collection was slide mounted following Winter (2000). The structure of the male genitalia did not allow the valves to be spread and flattened without damaging the sacculus, juxta, and transtilla. Whole specimens were cleared and stained following similar procedures as described for the genitalia.

Photographs of dissected specimens, genitalia, and adults were made using a Visionary Digital imaging system (http://www.visionarydigital.com, R. Larimer). Line drawings were made in Adobe Illustrator with a Wacom Intuos 4 drawing tablet from photographs or sketches made with a drawing tube attached to a Wild M5 stereomicroscope.

Eggs were obtained from gravid females collected in Box Canyon, Pima Co., AZ (18 July 2012). The females were placed in brown paper bags with Honey Mesquite (*Prosopis glandulosa* Torr.) foliage and bark. Larvae were reared on *Prosopis glandulosa* foliage.

## Systematics

### Heteranassa

Taxon classificationAnimaliaLepidopteraErebidae

Smith, 1899

#### Type species.

*Homoptera mima* Harvey, by subsequent designation by Nye 1975.

#### Taxonomy.

*Heteranassa* Smith, 1899: 105; Smith et al. 1903: 5; Barnes et al. 1917: 86; McDunnough 1938: 121; Kimball 1965: 130; Nye 1975: 239; Franclemont and Todd 1983; Poole 1989; Poole and Gentili 1996; Mustelin 2006: 7; Lafontaine and Schmidt 2010: 37; Zahiri et al. 2012: 118.

#### Diagnosis.

**Figure 2.**
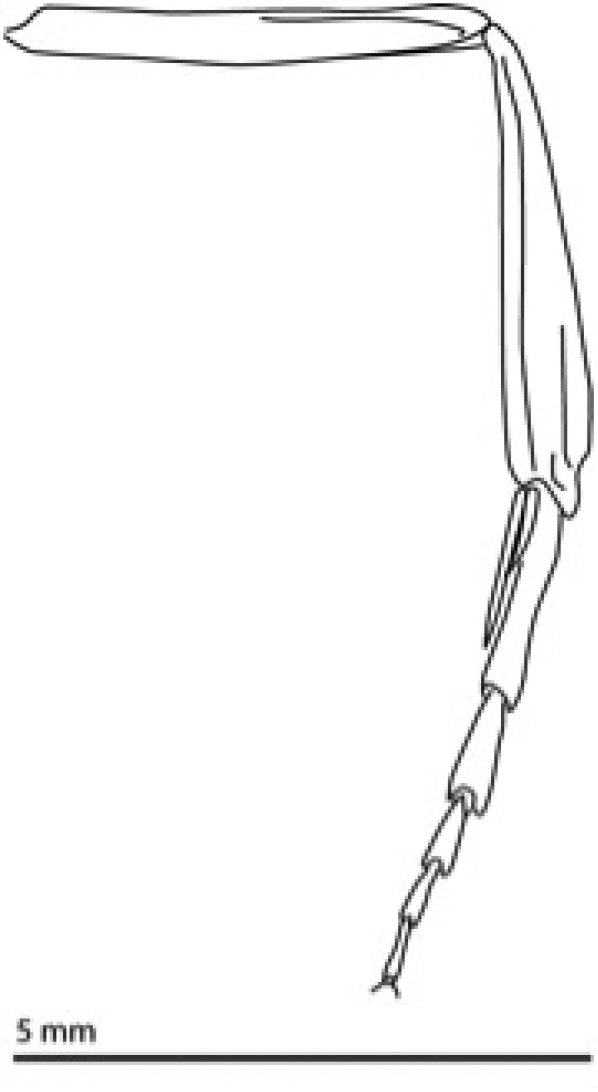
Middle leg of *Heteranassa mima*, showing middle tibia with spines absent.

**Figure 3.**
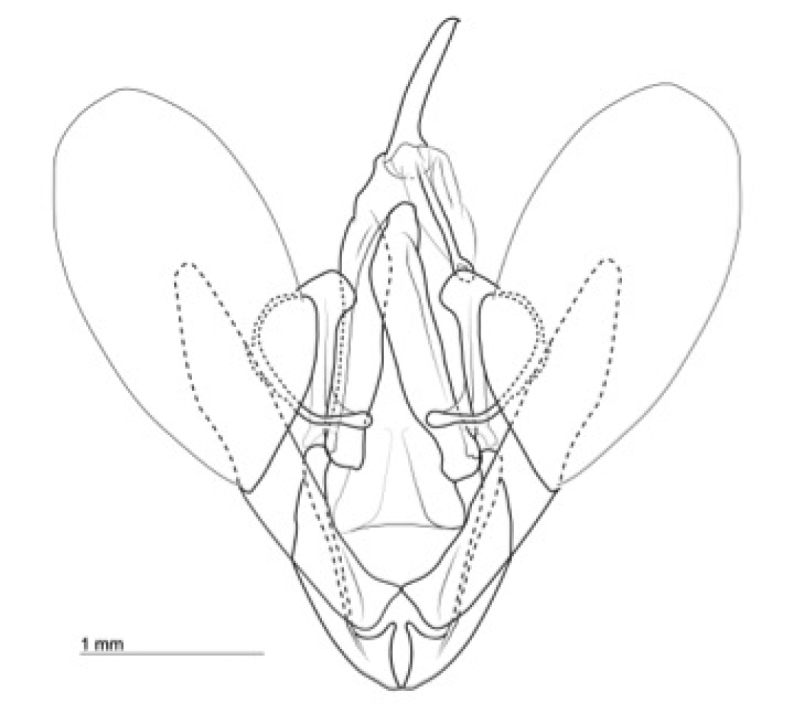
Valves, *Heteranassa mima*, ventral view.

**Figure 4.**
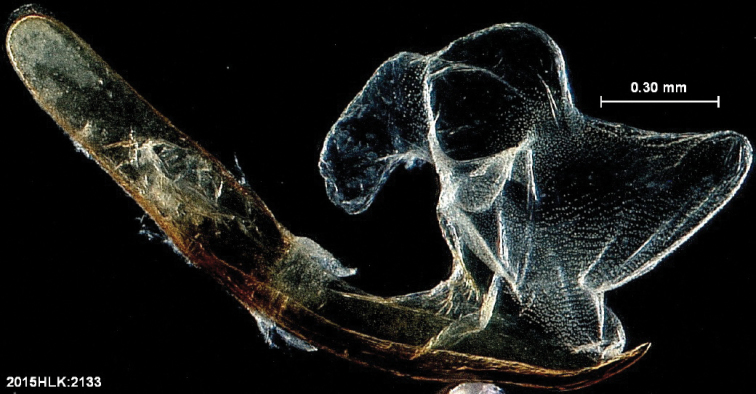
Aedeagus with vesica everted, showing diverticula.

**Figure 5.**
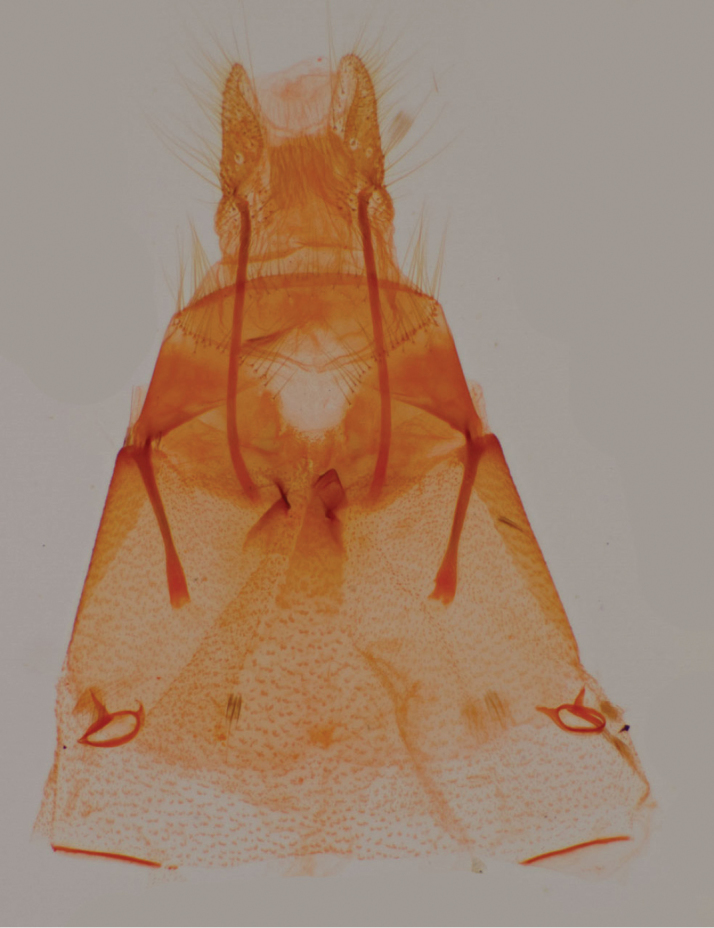
Female genitalia of *Heteranassa mima*.

**Figure 6.**
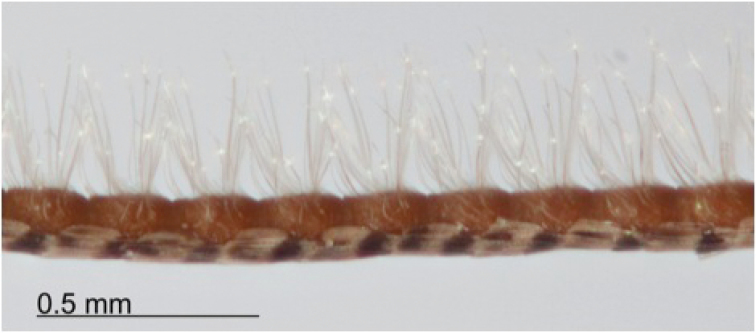
Fasciculate antenna of male *Heteranassa mima*.

**Figure 7.**
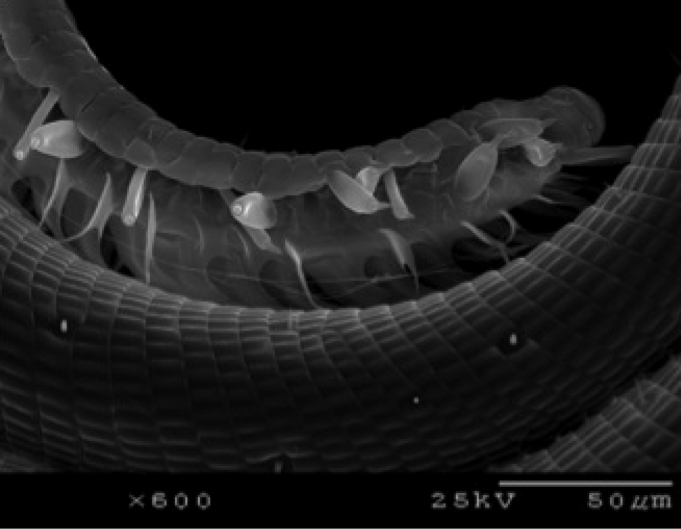
SEM of *Heteranassa mima* proboscis, showing sensilla styloconica.

*Heteranassa mima* is now the only valid species in the genus. The genus and species can be distinguished from similar genera by the absence of spine–like setae on the mesothoracic tibia (Fig. 2) (Smith 1899). The male genitalia (Figs 3, 4) serve to distinguish *Heteranassa* from other genera of Erebinae in the southwestern United States by the presence of a setose, membranous costal region of valves (Fig. 3) (Franclemont 1986), and a “D” shaped, sclerotized saccular process connecting to the saccular region of the valves (Fig. 3). The female genitalia (Fig. 5) does not differ dramatically from other Omopterini. Male antennae fasciculate (Fig. 6), female antennae filiform. The proboscis (Fig. 7) is well-developed.

Specimens of *Eubolina impartialis* Harvey, *Matigramma* species, *Acritogramma metaleuca* (Hampson), *Toxonprucha* species and *Coxina* species are frequently misidentified as *Heteranassa*. Of these, *Acritogramma metaleuca* is the most similar to *Heteranassa* (Franclemont 1986). *Acritogramma metaleuca* can be most easily distinguished by the presence of spine-like setae on the mesothoracic tibia, and there are also subtle differences in wing pattern (Franclemont 1986). *Acritogramma metaleuca* has no brown lines or shading on the forewing, and the discal spot is distinctly lunulate. *Eubolina impartialis* is similar to both *Heteranassa* and *Acritogramma metaleuca* but has a brownish ground color on the hindwings, instead of grayish white, and spine-like setae on the mesothoracic tibia. From southern Texas into Mexico, *Heteranassa* may be confused with co–occurring *Coxina* species. This genus shows affinities to *Heteranassa* in forewing pattern and genitalia, but a lighter hindwing ground color serves to separate *Heteranassa*. Additionally, similarities in wing pattern and genital morphology suggest a relationship to the Caribbean and South American genus *Elousa* Walker. The ranges of *Heteranassa* and *Elousa* may overlap in southern Mexico. *Elousa* can be separated from *Heteranassa* by its smaller size, and the light gray to white mottling of the forewings. *Toxonprucha* species are generally smaller than *Heteranassa*, and they possess hindwings with a darker ground color and more distinct patterning than those of *Heteranassa*. A key to *Heteranassa* and similar species is provided below.

#### Taxonomic history.

Harvey (1876) described *Homoptera mima* from a single female from Texas, listing Belfrage as the source of the specimen. He referred the species to the genus *Homoptera* Guenée, but did not mention any characters used to determine generic placement. Grote (1882), in a checklist, moved *Heteranassa mima* to the genus *Eubolina* Harvey, 1875, again without any mention of characters used. Smith (1899) described *Heteranassa fraterna* and *Heteranassa minor* and placed these species in the genus *Campometra* Guenée, 1852. *Campometra fraterna* was described from a series of six lightly–marked specimens collected in Death Valley, California., and a single specimen from Catalina Springs, Arizona. *Campometra minor* was described from a series of five small female specimens collected in Arizona. Smith described these two species as new based on differences in size, coloration, and patterning. Smith (1899) proposed the genus *Heteranassa* to circumscribe *Heteranassa mima*, *Heteranassa fraterna*, and *Heteranassa minor* based on the absence of spine–like setae on the mesothoracic tibia in these three species. Smith (1899) wrote, “I prefer leaving them with *Campometra* temporarily, until all of the allied genera can be carefully studied, but suggest the term *Heternassa* in case generic separation seems desirable.” These three species were formally referred to *Heteranassa* by Smith et al. (1903). Todd (1982) reviewed Smith’s type series and designated lectotypes for *Heteranassa minor* and *Heteranassa fraterna*. The pupa of *Heteranassa* was first described by Comstock (1955), and Crumb (1956) gave a description of the larva of *Heteranassa*.

In his study of southern California Noctuoidea, Mustelin (2006) determined *Heteranassa minor* to be a synonym of *Heteranassa fraterna*. He found no differences in genital morphology between the types of *Heteranassa fraterna* and *Heteranassa minor* (Mustelin 2006). Mustelin (2006) did not examine the type specimen of *Heteranassa mima*, located in the Natural History Museum, London.

#### Description.

**Figure 8.**
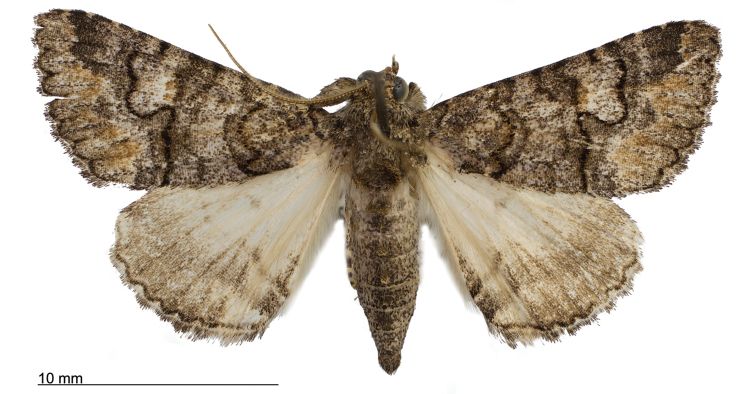
Male *Heteranassa mima*, showing complete FW maculation. Cuatrocienagas, Coahuila, Mexico, June.

**Figure 9.**
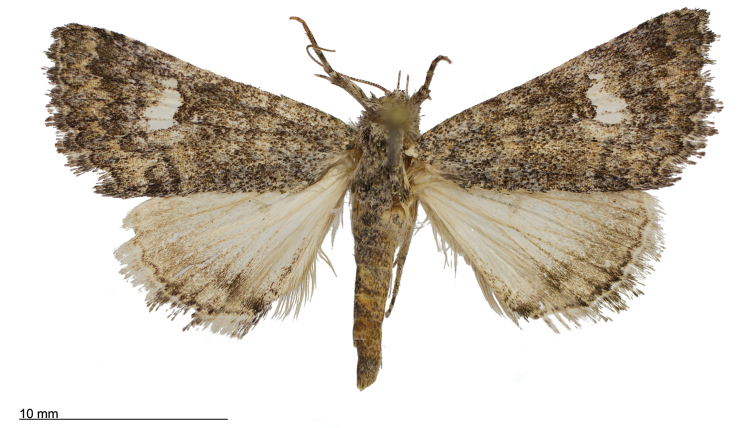
FW with dark ground color, white reniform spot, AM, medial, and PM lines very faint. Female *Heteranassa mima*, Inyo Co. CA, February.

**Figure 10.**
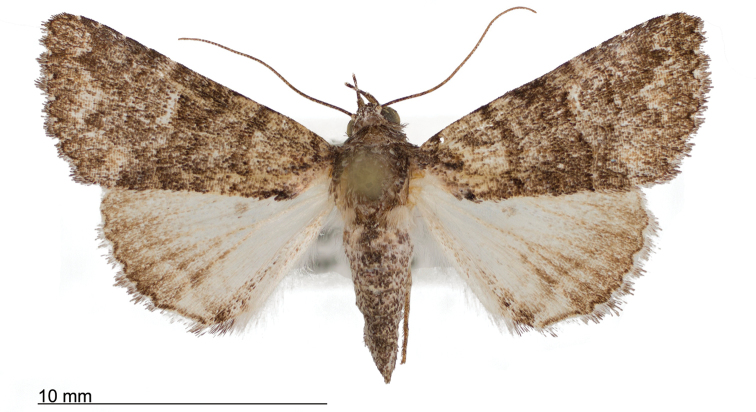
Thin, white dash in reniform, AM, medial lines visible, PM line extremely faint. Female *Heteranassa mima*, Maricopa Co. Arizona, March.

**Figure 11.**
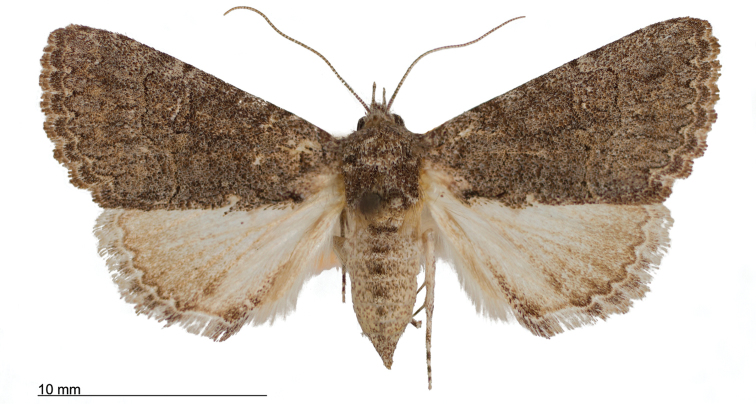
Thin, white reniform dash barely visible, PM line faint, AM, medial lines barely visible. Female *Heteranassa mima*, San Bernadino Co., California, April.

**Figure 12.**
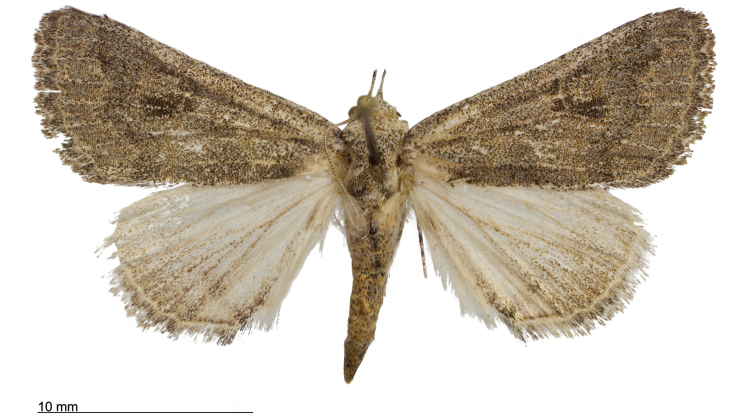
Tan FW ground color, AM, medial, PM lines absent. Female *Heteranassa mima*, Inyo Co., CA, February.

**Adult male** (Fig. 8): **Head**: front smooth scaled, vertex scales erect, elongate; labial palpi elongate, erect, three segments; area of frons behind labial palpi unscaled with domed center; antennae (Fig. 6) fasciculate, smooth scaled, conspicuous sensory setae on ventral surface; eyes smooth; proboscis well developed, coiled between labial palpi (Fig. 7). **Thorax**: smooth scaled dorsum; ventrally lighter; thick tuft of hairs arising below base of forewing. Legs: smooth scaled; prothoracic tibia with spatulate epiphysis, flattened hairs on ventral surface; mesothoracic tibia with thick tuft of scales, expanded distally, pair of spurs at distal end, spine-like setae absent; metathoracic tibia with pair of spurs mesially and at distal end; tarsi with three rows of spine-like setae. Forewing: 9.7–14.9 mm; antemedial line pointed apically on anal vein; medial line black, pointed mesially on radial, cubital, and anal veins; postmedial line black, outlining apical half of discal area; subterminal line brown, jagged, bordering lighter colored terminal area; terminal line scalloped outwardly at termini of veins, apical margin traced in lighter coloration; fringe scalloped apically at termini of veins; reniform spot markings range from white spot (Fig. 9), to thin white vertical dash (Fig. 10), to a barely visible dash (Figs 8, 11), or black (Fig. 12). **Hind wing**: ground color gray-white, darker shading distally; terminal line black, scalloped apically at termini of veins; fringe light gray, with dark shading between termini of M_3_ and CuA_2_ and between termini of 2A and 3A. **Abdomen**: segments 1 through 4 tufted dorsally. **Genitalia** (Figs 3, 4): Tegumen slightly excurved dorsally, lateral processes at distal end of each arm, process dorsally at distal end; uncus sparsely setose, curved, pointed; tuba analis membranous; scaphium sclerotized, tuba analis opening apically; juxta lightly sclerotized, excurved ventrally; transtilla membranous; vinculum U-shaped, mesial margin heavily sclerotized towards articulation with tegumen, widened in middle; valves conjoined basally, sclerotized basally, membranous distally; sacculus sclerotized; saccular process extended dorsally connected to membranous costal region; sclerotized part of valve with finger–like extension half distance from base; base of costa with a looped sclerite, connected to saccular process; aedeagus curved, narrowed apically, rounded anteriorly, dorsally sclerotized, ventrally membranous, dorsal surfaces undulating apically, apex pointed; ductus seminalis on ventral side; vesica membranous, without setae or cornuti, not elongated,four diverticula: one subbasal, two medial, and one apical. **Adult Female**: (Figs 7–10, 12) forewing length 11.0–16.7 mm. Exterior similar to male, except antennae filiform, mesothoracic tibiae not expanded distally. Genitalia: (Fig. 5) papilla analis membranous, rounded apically, setae stout, variable length; posterior apophysis extending just beyond anterior margin of 8^th^ abdominal segment, apically curved inwards; anterior apophysis ca. 0.5 × length of posterior apophysis, paddle-shaped apex; anal tube: interior lining of anal tube with many rows of minute spines directed anteriorly on dorsal wall, ventral wall densely covered with shark-tooth-like tubercles; intersegmental membrane with many shark-tooth-like tubercles; 8th abdominal segment ringed with stout setae caudally; ostium bursae lightly sclerotized; antrum circular, membranous; ductus bursae reduced, membranous; corpus bursae elongate, membranous.

**Eggs.** Dark bluish gray, ~1/2 mm diameter; captured females laid eggs singly or in groups of less than five in crevices of host plant bark, or singly on sides of enclosing container.

**Larvae.** Variable in color; eggs developed into adults within five weeks; observations are consistent with Comstock (1955) and Crumb (1956). Larvae pupated before high-quality photographs could be taken.

### Heteranassa mima

Taxon classificationAnimaliaLepidopteraErebidae

(Harvey, 1876)

Homoptera mima Harvey, 1876: 155–156.
Eubolina mima ; Grote 1882: 42; Smith 1891: 63; 1893: 372.
Campometra mima ; Smith 1899: 104–105; Dyar 1903: 237.
Elousa mima ; Draudt and Gaede (in Seitz) 1923: 478.
Heteranassa mima (Harvey, 1876); Smith et al. 1903: 5; Barnes et al. 1917: 86; McDunnough 1938: 121; Kimball 1965: 130; Franclemont and Todd 1983; Poole 1989, 1996; Mustelin 2006: 7; Lafontaine and Schmidt 2010: 37.
Campometra fraterna Smith, 1899: 104, **syn. n.**; Dyar 1903: 236.
Heteranassa fraterna (Smith, 1899); Smith et al. 1903: 5; Barnes et al. 1917: 86; McDunnough 1938: 121; Kimball 1965: 130; Franclemont and Todd 1983; Poole 1989, 1996; Mustelin 2006: 7; Lafontaine and Schmidt 2010: 37.
Elousa fraterna ; Draudt and Gaede (in Seitz) 1923: 478.
Campometra minor Smith, 1899: 104–105; Dyar 1903: 236.
Elousa minor : Draudt & Gaede (in Seitz) 1923: 478.
Heteranassa minor (Smith, 1899), **syn. n.**; Smith et al. 1903: 5; Barnes et al. 1917: 86; McDunnough 1938: 121; Kimball 1965: 130; Franclemont and Todd 1983; Poole 1989, 1996; Mustelin 2006: 7.

#### Diagnosis.

This is the only species in the genus and can be diagnosed with the generic combination (see above).

#### Type material.

**Figure 13.**
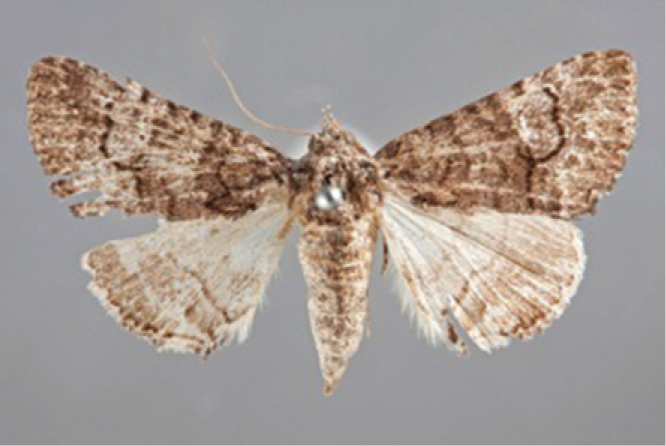
Holotype of *Heteranassa mima*, Texas.

***Heteranassa mima*** (Harvey, 1875). Holotype, (Fig. 13) ♀ in the Natural History Museum, London (BMNH) labeled: “Homoptera mima, type, Harvey, Holotype, 15/9, 73.” The specimen and associated labels were examined through high-resolution photographs provided by the BMNH. Type locality: Texas [USA]

**Figure 14.**
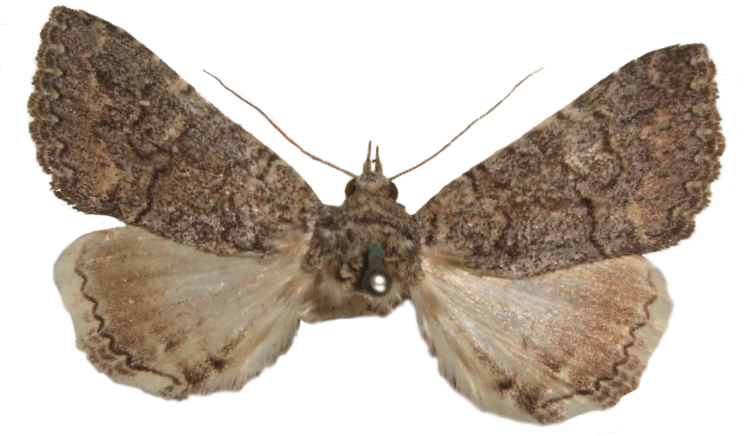
Lectotype of *Heteranassa fraterna*, Death Valley, California.

***Heteranassa fraterna*** (Smith, 1899). Lectotype (Fig. 14) ♀ in USNM, designated by Todd (1982), labeled: “Death Valley, April ‘91 K., 677, 115 [circled], ♀ genitalia on slide, Sept. 21, 1938, J.F.G.C. #2035, Type No. 4313 U.S.N.M., Lectotype, Campometra fraterna, Smith, Genitalia slide U.S.N.M. 40478, Campometra fraterna, ♀ Cotype, Smith” Type locality: Death Valley [California, USA]

**Figure 15.**
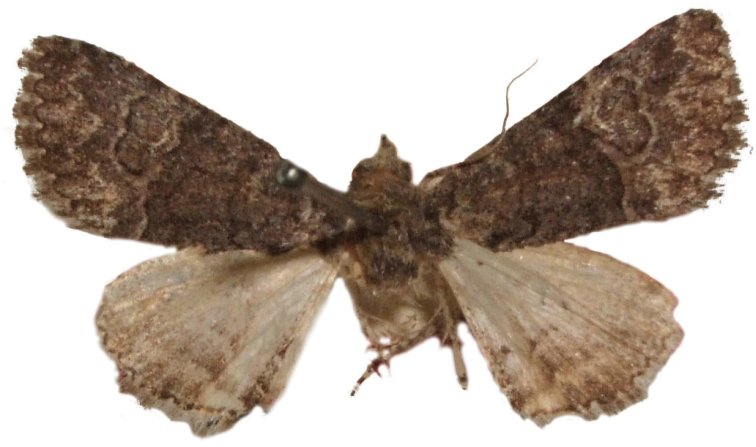
Lectotype of *Heteranassa minor*, Arizona.

***Heteranassa minor*** (Smith, 1899). Lectotype, (Fig. 15) ♀ in USNM labeled: “Campometra minor, ♀ type, Smith, Lectotype, Campometra minor, Smith, ♀ genitalia on slide, Sept. 21, 1938, J.F.G.C. #2035, Genitalia slide, U.S.N.M. 40477, Type No. 4314 U.S.N.M., U.S.N.M. Acc. no. 35005, Ariz., Collection G.D. Hulst.” Type locality: Arizona [USA]

#### Description.

**Adult male** (Fig. 8): **Head**: scaling dark gray to gray-brown to tan; alternating uneven banding of white to light brown scales, and dark-brown scales, labial palpus concolorous with head and body, antenna scaling: each segment alternating light gray and dark brown. **Thorax**: dorsum dark gray to gray brown to tan; venter lighter grayish brown. Legs: dorsally concolorous with thorax, ventrally light gray with darker scales, tarsi alternating white and dark brown; tarsal segments alternating dark–brown to white scaling. Forewing: length as for genus description, basal line black; band of darker color runs vertically, adjacent to antemedial line, terminating where antemedia line points apically; area between medial and postmedial lines shaded darker, excluding reniform area; crenulations on margin of forewing with gray-white punctations. Hind wing: shaded gray brown from medial area distally; postmedial line complete, or faintly visible distally; subterminal line darker gray brown, outlined with light coloration distally. **Abdomen**: dorsum dark gray to gray brown to tan, laterally gray; venter gray, dusted with darker scales. **Genitalia** (Figs 3, 4) (24 dissections): Lateral processes at distal end of tegumen arms wavy; process at dorsal end fin shaped, very weakly sclerotized; ventral membrane on distal end weakly sclerotized; juxta with numerous short, pointed tubercles mesially, narrowed caudally; transtilla attached to costal parts of valve processes; vinculum with flared, fin-like processes directed anteriorly; sclerotized saccular process looping, connecting to costal region, “D” shaped; base of costa thumblike, connected to transtilla; aedeagus with dorsal surface undulating apically; vesical with five diverticula. **Adult Female** (Figs 9–15): forewing length as in genus description. Similar to male, except antennae filiform. **Genitalia** (Fig. 5) (12 dissections): Postvaginal plate narrowed anteriorly, densely covered in shark-tooth-like tubercles, caudal 7/8^th^ outlined in stout setae; sterigma with sclerotized ridges laterally.

#### Variation.

Specimens tend to be larger in the eastern part of the range in Texas, and smaller specimens are more common in Arizona and California. Forewing coloration ranges from dark gray with some brown dusting to tan. Maculation ranges from well–defined antemedial, postmedial, and subcostal lines to lightly marked specimens with only the subcostal line well defined. Lightly marked specimens are found most commonly in the Mojave Desert. Ground color of hind wings is lighter towards the western range of the species. Specimens from the eastern part of the range show distinctly marked discal spots and shading on the margins on ventral surface of the wings, and the undersides are more heavily dusted with darker scales. The size of the white patch in the reniform area varies from a narrow dash to a large spot, while forewing ground color ranged from dark gray to gray brown among moths reared from the same female collected in Southeast Arizona.

Barcode variation in *Heteranassa* is very conservative. Examination of more than 160 full-sequence (658 base-pair) barcodes from California, Arizona, New Mexico, Texas, and northern Mexico showed a maximum divergence of less than 0.8%. One haplotype^*^ dominated the sample, representing more than half of the specimens; the other barcodes included 36 haplotypes that had no more than two base-pair differences from each other. One haplotype, restricted to central and southern Texas, departed from this pattern in being 0.8% different from those from farther west. This is most probably the haplotype that should be associated with the name *Heteranassa mima*, it being described from this part of Texas. However, this “eastern” haplotype is found with “western” haplotypes in central Texas and there is no indication in genital structural characters, or wing color or pattern, that *Heteranassa* includes more than a single species. The barcodes of *Heteranassa* are so divergent that they give no indication of a close relationship to any other erebid genus, other than belonging in the subfamily Erebinae, tribe Omopterini. *Heteranassa* specimens from Texas and Mexico are frequently confused with some species associated with the genus *Coxina* Guenée, which can have a similar superficial pattern, but the barcodes are more than 10% different and the two genera do not appear to be closely related. (D. Lafontaine pers. comm.).

^*^CNCNoctuoidea13382 [Baboquivari Mts., Pima Co., Arizona, USA]

AACTTTATATTTTATTTTTGGAATTTGAGCAGGAATAGTAGGAACCTCTTTAAGTTTATTAATTCGTGCTGAATTAGGAAACCCTGGTTCTTTAATTGGAGATGATCAAATTTATAATACTATTGTTACAGCTCATGCTTTTATTATAATTTTCTTTATAGTTATACCAATTATAATTGGAGGATTTGGAAATTGATTAGTCCCCTTAATATTAGGAGCTCCTGATATAGCTTTCCCTCGAATAAATAATATAAGTTTCTGATTATTACCCCCATCTTTAACTCTTTTAATCTCAAGAAGAATCGTAGAAAATGGAGCAGGAACAGGATGAACAGTTTACCCCCCACTTTCATCTAACATTGCTCATAGAGGAAGATCAGTAGATTTAGCAATTTTCTCTCTTCATTTAGCTGGAATTTCATCAATTTTAGGAGCTATTAATTTTATTACTACTATTATCAATATACGATTAAATAGATTAATATTTGACCAAATACCTTTATTTGTTTGAGCTGTTGGTATTACTGCTTTTTTACTATTATTATCTTTACCTGTTTTAGCTGGAGCTATTACTATACTCTTAACAGATCGAAATTTAAATACTTCCTTTTTTGATCCTGCTGGAGGAGGAGATCCTATTCTTTACCAACATCTATTT

#### Distribution and habitat.

**Figure 16.**
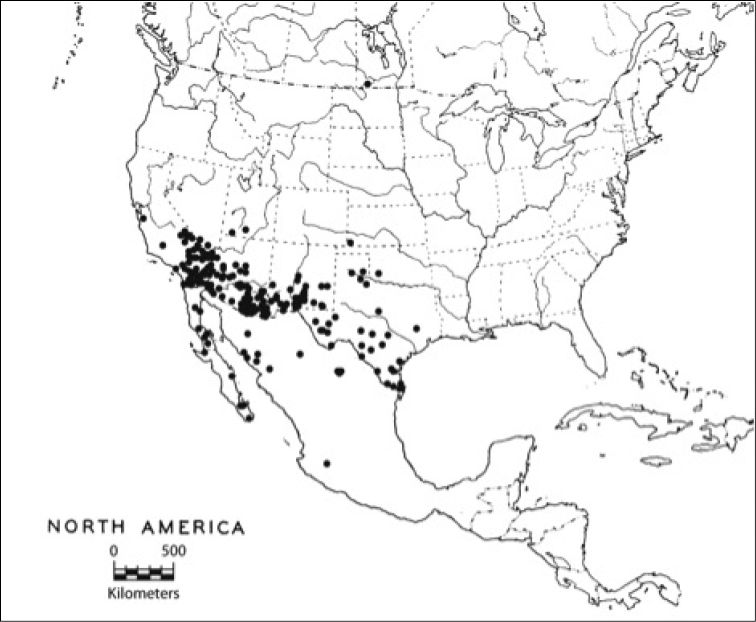
The distribution of *Heteranassa* in North America, ranging from Texas and Oklahoma west to California, USA, south to Jalisco Mexico. A single stray is recorded from Manitoba, Canada.

Warm, arid habitats from California to Texas, northward to Oklahoma, and south as far as Oaxaca, Mexico (Fig. 16). A single specimen from Cartwright, Manitoba is in the LACM.

#### Discussion.

The variation in *Heteranassa* wing pattern and coloration is continuous, with many specimens appearing intermediate to the phenotypes described by Smith (1899) and Harvey (1876). Genitalic morphology does not, however, correlate with wing pattern differences. These observations suggest that *Heteranassa* contains a single, highly variable species, *Heteranassa mima*. Studies of another erebine genus, *Catocala*, have shown that pressure from avian predators may drive high levels of polymorphism in forewing pattern and coloration (Ricklefs and O’Rourke 1975, Bond and Kamil 2002, Webster et al. 2009), and *Heteranassa* may be subject to similar evolutionary processes.

A series of *Heteranassa* from Death Valley, California collected in February, 2005, is the most variable in forewing pattern and coloration among the thousands of specimens observed to date. *Heteranassa* comprised roughly 90% of the moth specimens collected during this period, demonstrating that the genus is an abundant and likely ecologically important insect herbivore in North American desert biomes.

During the course of this research, we became aware of potential taxonomic affinities with the neotropical genera *Elousa* Walker (1857) and *Coxina* Guenée (1852). These genera have not been studied in a systematic framework since the turn of the 20th Century. A preliminary examination of male genitalia and wing pattern show significant overlap of characteristics between the genera. These three genera lack spines on the mesothoracic tibiae, and possess symmetrical male genitalia with membranous costal regions of the valves. These processes are larger in *Heteranassa* and *Elousa albicans* (Walker, 1857) than they are in *Elousa schausi* (Giacomelli, 1911) and the other *Coxina* species we have dissected. We have examined 10 species in these genera from the Caribbean and South America. Specimens belonging to this group that we collected in the Nicaraguan highlands appear more similar to *Elousa schausi* specimens from Argentina and *Coxina* specimens from Mexico, south Texas, and Florida, than they do to Caribbean *Elousa* or North American *Heteranassa*. Future research could test the monophyly of *Coxina* and *Elousa* with respect to *Heteranassa*, and how these genera speciated in North and South America and the Caribbean.

### Key to *Heteranassa* and similar species in the southwestern United States

**Table:** 

1	Hindwing and forewing with similar coloration and patterning	***Matigramma***, ***Toxonprucha***
–	Hindwing with different coloration and patterning than forewing	**2**
2	Ground color of hindwings chocolate to dark brown	**3**
–	Ground color of hind wings light gray to white, with some darker scaling towards the margins	**4**
3	Middle tibia with spine-like setae	***Eubolina***
–	Middle tibia without spines	***Coxina***
4	Middle tibia with spines	***Acritogramma metaleuca***
–	Middle tibia without spines	***Heteranassa***

## Supplementary Material

XML Treatment for Heteranassa

XML Treatment for Heteranassa mima

## Supplementary materials

Supplementary material 1 Table S1. List of examined Heteranassa specimens. Data type: occurence Explanation note: Contains localities of all specimens examined in this study. This dataset is made available under the Open Database License (http://opendatacommons.org/licenses/odbl/1.0/). The Open Database License (ODbL) is a license agreement intended to allow users to freely share, modify, and use this Dataset while maintaining this same freedom for others, provided that the original source and author(s) are credited.Nicholas Homziak, Heidi Hopkins, Kelly B. Miller

Supplementary material 2 Table S2. List of dissected Heteranassa specimens. Data type: occurence This dataset is made available under the Open Database License (http://opendatacommons.org/licenses/odbl/1.0/). The Open Database License (ODbL) is a license agreement intended to allow users to freely share, modify, and use this Dataset while maintaining this same freedom for others, provided that the original source and author(s) are credited.Nicholas Homziak, Heidi Hopkins, Kelly B. Miller
